# A modification in Weibull parameters to achieve a more accurate probability distribution function in fatigue applications

**DOI:** 10.1038/s41598-023-44907-9

**Published:** 2023-10-16

**Authors:** H. Fakoor, J. Alizadeh Kaklar

**Affiliations:** Urmia University, Urmia, Iran

**Keywords:** Mechanical engineering, Mechanical properties

## Abstract

Risk evaluation for fatigue failure of the engineering components is an important aspect of the engineering design. Weibull distributions are often used in preference to the log-normal distribution to analyze probability aspects of fatigue results. This study presents a probabilistic model for calculating Weibull distribution parameters to reduce the effect of percentage discretization error of experimental fatigue life and R–S–N curves for three reliability levels. By considering any result of standard fatigue test as an equivalent Weibull distribution, artificial data are generated and the accuracy of common Weibull distribution model can be improved. The results show error reduction in the Kolmogorov–Smirnov test and R-square values. Also, the Basquin model is used for different reliability levels with the same error order for risk evaluation of fatigue failure. The coefficient of variation for fatigue life increases at higher stress levels and has a linear relation with stress level for a high-cycle fatigue regime.

## Introduction

Fatigue failure is the formation and propagation of cracks due to a repetitive or cyclic load. It has been estimated that fatigue contributes to approximately 90% of all mechanical service failures^1^. For the first time, the French mathematician and engineer Jean-Victor Poncelet used the terminology of “fatigue” in his book in 1841^2^. For fatigue base designs, actual fatigue data should be used or, if not available, must be modeled and generated. Many models have been developed in the S–N (stress-life) approach of fatigue life estimation to depict S–N curves. The earlier models have estimated a median fatigue life, but it is necessary to calculate the risk of fatigue failure for a safe and economical design. The fatigue life shall be predetermined by the desired reliability levels referred to by the material properties, crack size, environment, and loading condition. In engineering designs, the failure of system parts must be considered with a low probability of occurrence^3^.

In statistical analyses, probabilistic fatigue S–N curves are widely used to quantify scattered fatigue test data and analyze fatigue problems. Usually, for constructing the curves, fatigue tests are performed on some stress levels. Then, a distribution model (e.g., Weibull) is applied to predict fatigue life at a specified applied stress level, followed by characterizing the S–N relation. The Basquin equation is an S–N relation with a linear relationship between applied stress and fatigue life logarithmic scale. This equation can be used to express the stress-life relation in a certain survival probability confidence level. This probabilistic S–N (R-S–N) curve is an S–N relation in some probability confidence levels^4^.

This paper focuses on a modification for a common probability distribution model of a physical phenomenon when the available number of experimental results is limited. The importance of this idea is that generally, engineering design costs account on average for 5% of total costs in the projects^5^ and most of the cost is related to conducting experimental tests. Weibull distribution is often used in preference to analyze probability aspects of fatigue results^6^ and to reduce required number of experimental tests for evaluation of fatigue life distribution. The novelty of this paper is making some artificial data by considering any result of the fatigue test as an equivalent Weibull distribution with the mean value of the same test fatigue life and cumulative probability of common Weibull method. The accuracy of common Weibull distribution model can be improved using this method.

### Probability distributions

Probability theory is one of the most important aspects of statistics. A probability distribution is a mathematical function that gives the probabilities of occurrence of different possible outcomes for an experiment^7^. Table 1 presents several models that have been proposed to model probability distribution.

**Table 1:** Common models representing a probability distribution.

Model	Equation
Normal distribution or Gaussian distribution	$f\left(x\right)=\frac{1}{\sigma \sqrt{2\pi }}{e}^{{-\frac{1}{2}\left(\frac{x-\mu }{\sigma }\right)}^{2}}$
Pareto distribution	$$F\left(x\right)=\left\{\begin{array}{ll}1-{\left(\frac{{x}_{m}}{x}\right)}^{\alpha }, & x\ge {x}_{m}\\ 0, & x<{x}_{m}\end{array}\right.$$
Continuous uniform distribution	$$\normalsize F\left(x\right)=\left\{\begin{array}{ll}0, & x-\mu <-\sigma \sqrt{3}\\ \frac{1}{2}\left(\frac{x-\mu }{\sigma \sqrt{3}}+1\right), & -\sigma \sqrt{3}\le x-\mu <\sigma \sqrt{3}\\ 1, & x-\mu \ge \sigma \sqrt{3}\end{array}\right.$$
Weibull distribution	$F\left({N}_{f}\right)=1-{e}^{-{\left(\frac{{N}_{f}}{\theta }\right)}^{b}}$
Gumbel distribution	$F\left(x;\mu ,\beta \right)={e}^{-{e}^{-(x-\mu )/\beta }}$
Tukey lambda distribution	$$Q\left(p;\lambda \right)=\left\{\begin{array}{ll}\frac{1}{\lambda }\left[{p}^{\lambda }-(1-p{)}^{\lambda }\right], & \lambda \ne 0\\ log\left(\frac{p}{1-p}\right), & \lambda =0\end{array}\right.$$
Exponential distribution	$$f\left(x,\lambda \right)=\left\{\begin{array}{ll}\lambda {e}^{-\lambda x}, & x\ge 0\\ 0, & x<0\end{array}\right.$$

The probability distributions are described with statistical parameters like mean and standard deviation, as shown in Table 1. Normal distribution, named Gaussian distribution, is the most frequently used distribution function in statistical analysis. The normal distribution, which has a bell-shaped curve, has been used for independent, random variables in the survey reports, Technical Stock Market, and scientific study of many observable phenomena in nature like human height or IQ distribution. Sinclair and Dolan^8^ conducted a comprehensive statistical fatigue investigation engaging 174 nominal identical, extremely polished, smooth 7075-T6 aluminum alloy specimens. They worked on 6 alternating applied stress levels at the fully reversed test conditions. A normal distribution in the logarithmic scale for the experimental results at every applied stress amplitude gives the impression of being reasonable. Derived from that and the other performed statistical fatigue test results, a logarithmic scale normal distribution of failure is usually considered in fatigue analysis. At every applied stress range, a group of S–N diagrams at different percentages of failure probabilities are established from the probability distribution functions.

The Pareto distribution is one of the power-law probability distributions in probability theory and mathematics. Pareto distribution is usually used in the explanation of data scatter in the many types of scientific studies. Particularly applied to describing the distribution of wealth in a society, fitting the trend that a large portion of wealth is held by a small fraction of the population^9^.

The Gumbel distribution usually has been applied to figure the extremum (Max or Min) distribution of a number of samples of different statistical societies. Forecasting a temblor, torrent, or other types of natural disaster is of great use and value. For instance, if the database of a parameter of a phenomenon like water in the river for 10 years ago is available, Gumbel distribution can be applied to predetermine the distribution of the variation of extremum of water level at a river in the specific time. For the Gumbel distribution, the special importance is to predict the extremum distribution according to extreme value theory^10^.

John Tukey presented a continuous symmetric probability distribution model in which the Tukey lambda distribution function was specified in terms of its quantile function. The Tukey lambda distribution function is used to recognize a suitable distribution. Therefore, the Tukey lambda distribution usually has no direct application in statistical models. The Tukey lambda distribution has a single shape parameter that can be rearranged and defined in terms of the standard distribution.

The exponential distribution is a continuous distribution for modeling events that occur at a constant time rate. Two important applications of the exponential distribution are the modeling of radioactive decay in physics and the modeling of the posterior default probability for a set of financial assets in finance^11^. The exponential distribution can be applied to analyze the relationship between the unobservable actual values and measurement values^12^. In some cases the lifetime of a manufacturing item may fallows a mixed distribution models such as the half-normal distribution and the half-exponential distribution^13^.

Among the probability distributions used to analyze fatigue problems^14,15^, the Weibull distribution is one of the most common models in the logarithmical scale. Weibull progressed a new approach and used it to study fatigue actual data^16,17^. Weibull distributions have 2-parameter and 3-parameter models. The 2-parameter model is widely extended in fatigue problems and design. In this approach, the expected fatigue life range starts from zero cycles. Indeed, the 2-parameter Weibull distribution is a simplified 3-parameter Weibull distribution with a minimum expected life of zero. At the same time, the 3-parameter distribution is characterized by a finite minimum life greater than zero. For 3.3 ≤ b ≤ 3.5, the Weibull distribution function is approximately normal or Gaussian, while it is exponential for b = 1. The coefficient of variation (standard deviation/mean) is approximately C = l/b for the two-parameter Weibull distribution. For b values between 3 and 6, (typical of fatigue), the error from this approximation is about 10 to 15%^6^. In modern life testing analysis to obtain information about fatigue life of a component, new method of experimental process is conducted, where products are tested under higher stress than normal to get their failure information. For example, new methods such as adaptive type-I progressive hybrid censoring is planned to evaluate the failure parameters assuming that the failure causes are independent Weibull variables^18^.

In a study of statistical fatigue analysis, Zhao and Liu^4^ proposed a Weibull approach to the probabilistic study. They investigated stress-life for rolling contact fatigue. The study shows that the 2-parameter and 3-parameter Weibull equations have reasonable results. However, the 3-Parameter Weibull model has a lower standard deviation for fatigue life. This standard deviation decreases at higher applied stress levels. These results are consistent with those of classic fatigue studies.

Xionga et al.^19^ investigated multiaxial fatigue results of magnesium alloy using the modified Smith–Watson–Topper (SWT) theories and the multiaxial Jiang criterion. The results of both theories were acceptable. Jiang et al.^20^ used the Markov chain Monte Carlo method to estimate the parameters of a modified Weibull distribution. They suggested the use of Markov chain Monte Carlo estimation instead of maximum-likelihood estimation for point estimation when the sample size is less than 100. Canteli et al.^21^ studied 3 types of fatigue models, namely, LCF, HCF, and VHCF, which are usually used in mechanical parts design. The study presented the actual results of the stress-based and strain-based approaches in a single methodology. Strzelecki^22^ used 2-P and 3-P Weibull distribution and presented features of the S–N curve for fatigue limit investigation. The fatigue test results were used for rotary bending of S355J2 + C and C45 + C steels, and the S–N curves were specified. Acosta et al.^23^ used measurement techniques based on temperature and magnetics to describe the fatigue behavior of metallic materials. Furthermore, they reduced the effort required to generate and provide S–N curves using valuable input parameters for short-time fatigue life calculation methods.

### S–N Relation

In the S–N approach, many models have been developed for evaluating the S–N relation, with some shown in Table 2. Basquin^24^ suggested a linear relation in the logarithmical scale between the applied stress (S) and the fatigue life (N). Basquin’s equation is generally developed in the standards such as ASTM, ASME, and UNE and Guidelines such as FKM, DNV, and GL. Vidovic^25^ performed an analytical study of the maximum-likelihood estimations for the parameters of a modified Weibull distribution model and indicated that their implementation in practice follows a rather simple pattern. Usabiaga et al.^26^ implemented a model on the NCode2020 software to demonstrate the probable implementation in general commercial codes by major applications on fatigue design.

**Table 2:** Common methods to estimate the S–N curves^24^.

Model	S–N curves relation
Basquin (1910)	$\log N=A-B\ \log(\Delta \sigma) ;\ \Delta \sigma \ge \Delta {\sigma }_{\infty }$
Stromeyer (1914)	$\log N=A-B\ \log(\Delta \sigma -\Delta {\sigma }_{\infty })$
Palmgren (1924)	$\Delta \sigma =b(N+B{)}^{-a}+\Delta {\sigma }_{\infty })$
Bastenaire (1972)	$N=\frac{A}{\Delta \sigma -E}\mathrm{exp}\left[-C\left(\Delta \sigma -E\right)\right]-B$
Ling and Pan (1997)	$F=\sum_{i=1}^{n}\left\{\mathrm{In\ }(\sigma\left({S}_{i}\right))+\frac{[ \log{N}_{i}-\mu ({S}_{i}{)]}^{2}}{2{\sigma }^{2}({S}_{i})}\right\}$
Kohout and Vechet (2001)	$\log\left(\frac{\Delta \sigma }{\Delta {\sigma }_{\infty }}\right)=\mathrm{log}{\left(\frac{{N+N}_{1}}{{N+N}_{2}}\right)}^{b}$

Crack propagation has always been a source of concern in determining inspection routines in different industries. Crack propagation at the higher applied stress amplitude can cause great uncertainty in the fatigue life estimation. Crack propagation has been studied widely to characterize different types of cracks, including edge, surface, subsurface, etc.^27–29^. Focus on these studies shows the uncertainty and wide data scatter in the fatigue life compared to other mechanical properties may be due to diversity in the crack initiation location and different crack types.

Stromeyer^30^ published the empirical relation for the mathematical description of fatigue. Basquin, in his fatigue relation, had not considered the idea of the fatigue limit. Stromeyer^30^ studied Wöhler’s fatigue test data. To verify the existence of the fatigue limit concept, they conducted advanced rotating-beam fatigue tests on several materials to verify the existence of a definite fatigue limit. The Stromeyer law represents the Stress-Life curve by truncating the Basquin relation at the fatigue limit by plotting the load and fatigue life. In addition, Stromeyer presented a relation between fatigue samples temperature increase and fatigue limit. However, the knee point (*N*_knee_)^31^ was not specified explicitly.

Palmgren^32^ presented a new theory resembling the Basquin method and the Stromeyer method. The equation in Table 2 presents the results of the fatigue test of rolling bearings. The fundamental relation of this method involves the stressed volume of material in the rolling bearing raceway sub surfaces as the main parameter. “This volume of material is simplistically determined to have a nearly rectangular subsurface cross-sectional area bounded by the length of the maximum contact area ellipse and the depth at which the maximum failure-causing stress occurs”.

In fatigue design, an adequate quantification of ISO 12107 inherent variation is one of the essential parameters for calculating the fatigue property in the various mechanical parts of systems and components. Also, it is essential to compare materials in fatigue properties, including their variation in engineering design. In this respect, statistical methods have been used widely to compare material properties. This International Standard includes a full methodology for the application of the Bastenaire model as well as other more sophisticated relationships. It also addresses the analysis of runout (censored) data^33^. Ling and Pan^34^ presented a new method to determine R–S–N curves to minimize the cost and the number of samples needed for laboratory testing. The stress-life curves were considered in a 3-parameter form.

Kohout and Vechet^35^ presented a different method to define S–N curves in the whole cyclic load domain in fatigue problems. This method incorporates all the fatigue-affected regions from ultimate strength of material to fatigue endurance limit, which is generally expressed by the Palmgren function. For every region, this method is similar to one of the previous theories, i.e., when the applied load is approximately large, the Kohout and Vechet model converts into the Basquin model. On the other hand, when the applied load is smaller than the fatigue endurance limit, this model converts to the Stromeyer function for almost infinite life and high-cycle fatigue region. Compared to the models specified above, the Kohout and Vechet model has some precedence. This method has a better curve fitting of fatigue test results, and this coefficient has unambiguous technical and geometrical meaning, which can be calculated with higher accuracy. In addition, this model is more appropriate for extrapolation and interpolation for fitted curves in the low-cycle and very-high-cycle regions.

The present work tries to develop the probabilistic S–N relationships for existing fatigue data in the following three steps: (1) collecting the fatigue test data; (2) estimating the probabilistic curves for every specified test condition (the key task in the present work is to determine the Weibull equation coefficient for scattered test data); and (3) evaluating the Basquin equation’s coefficient from the previous step’s data (to this end, a regression analysis will be done on the estimated fatigue life from the previous step).

## Formulation of modified Weibull approach

### Life distribution

#### Tolerance limits

Fatigue data are subjected to considerable scatter. In statistical analysis, a sample with a random data set is chosen. Obtaining data from the entire population is usually impossible or has very high undue costs. Due to sample size limitations, the sample statistical parameters, including mean median or variance values, are different from the source population. Designating a confidence level assigns a quantitative value of uncertainty or confidence. Lower and upper tolerance limits in a Weibull distributed model can be calculated using Eq. (1a) and (1b)^6^:

1a $$\mathrm{Lower \, limit}=\mathrm{F}\left({N}_{f}\right)-k$$

1b $$\mathrm{Upper \, limit}=\mathrm{F}\left({N}_{f}\right)+k$$

where $k$ is a function of the sample size.

Replacement of sample statistical properties with source population properties involves some degree of uncertainty. This uncertainty is determined using the percent error. If the sample average is x_1_, the percent error will be^4^:

2 $$\mathrm{Percent \, error}=\pm \left[\frac{{s}_{1}{t}_{\frac{\alpha }{2};\vartheta }}{{\overline{X} }_{1}\sqrt{n}}\right]$$

where ${\overline{X} }_{1}$ is the sample logarithmical average life, n is the sample size, $\vartheta$ is the degree of freedom, $\frac{\alpha }{2}$ is the degree of confidence, and confidence is equal to $(1-\alpha$), ${S}_{1}$ is the logarithmical standard deviation of sample life. Note that the value of t statistics is available in standard tables.

The present study reviewed the effect of stress level on the data scatter and coefficient of variation. S_1_ and ${\overline{X} }_{1}$ values were evaluated in the linear scale.

#### Probability distributions of samples

For the evaluation of the distribution of each sample data set, two-parameter and three-parameter Weibull distribution functions can be established as^6^:

3 $$F\left({N}_{f}\right)=1-{e}^{-{\left(\frac{{N}_{f}-{N}_{{f}_{0}}}{\varnothing -{N}_{{f}_{0}}}\right)}^{b}}$$

where $\mathrm{F}\left({N}_{f}\right)$ is the failure fraction in the test data set ${N}_{f}$, ${N}_{{f}_{0}}$ is the minimum expected fatigue life, $\theta =\varnothing -{N}_{{f}_{0}}$ is characteristic fatigue life (cycles when 63.2% have failed), and $b$ is the Weibull slope or shape parameter. The terms ${N}_{{f}_{0}}$, $\theta$, and $b$ are 3-parameter Weibull model, and for the 2-parameter Weibull model, the parameter of ${N}_{{f}_{0}}$ is zero, ${N}_{{f}_{0}}=0$.

To determine the Weibull equation coefficient for scattered test data (which is the key task in the present work), a step function, i.e., $(i-0.3)/(n+0.4)$ is commonly used as a percent of failure. In the present work, every failure life of ${N}_{{f}_{i}}$ in test results is considered as a Weibull distribution with a median value of ${N}_{{f}_{i}}$ and cumulative probability of $(i-0.3)/(n+0.4)$. The failure fraction for median value of ${N}_{{f}_{i}}$ in Eq. (3) will be 50%, so characteristic fatigue life, ${\theta }_{i}$, the minimum expected fatigue life, $\gamma$ and the Weibull slope, $b$ for any failure life, ${N}_{i}$ will be:

4a $$\mathrm{for }\ i=1\ to\ n: 0.5={e}^{-{\left(\frac{{N}_{i}-\gamma }{{\theta }_{i}}\right)}^{b}}$$

Then:

4b $${\theta }_{i}=\frac{{N}_{i}-\gamma }{\sqrt[b]{-\mathrm{ln}(0.5)}}$$

The Weibull equation for every test result from Eq. (4b) is:

4c $${P}_{i}(N@{S}_{ca})={e}^{-{\left(\frac{{N}_{i}-\gamma }{{\theta }_{i}}\right)}^{b}}$$

where ${P}_{i}(N@{S}_{ca})$ is the expected failure fraction for fatigue life $N$ at applied stress level of ${S}_{ca}$. By applying Eq. (4c) and weight factor, the modified Weibull function is derived:

5 $${P'}\left({N}_{j}@{S}_{ca}\right)=1-\sum_{i=1}^{n}\frac{1}{n}{\int }_{0}^{{N}_{j}}(1-{P}_{i}\left(N@{S}_{ca}\right))dN$$

**Figure 1:**
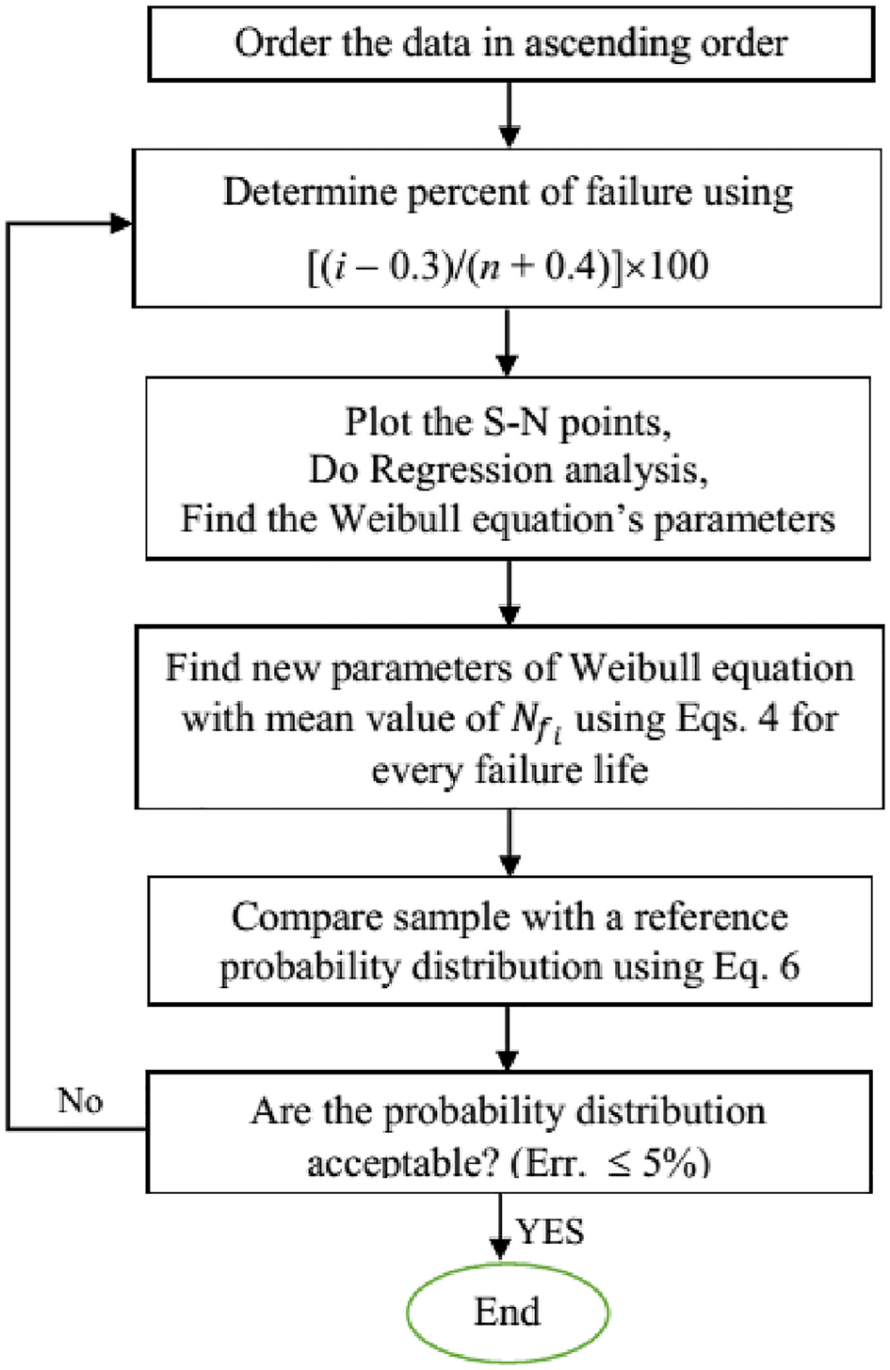
Flowchart of parameters calculation for Modified Weibull distribution.

Weibull equation coefficients are determined based on the flowchart in Fig. 1. First, Weibull parameters will be calculated by the common Weibull model (Stages 1 to 3 in the flowchart). Then, for every single test data, an equivalent Weibull distribution with the mean value of the same test fatigue life and cumulative probability of common Weibull method and modified Weibull parameters will be calculated using Eqs. (4a), (4b), (4c) and (5) (Stages 4 and 5 in the flowchart). Finally, test data were compared with calculated parameters and presented probability distribution model using the Kolmogorov–Smirnov test (K–S test), as follows (Stages 6 and 7):

6 $${D}_{i}=ma{x}_{i=1}^{k}\left[\left|{F}^{*}\left({x}_{i}\right)-F({x}_{i})\right|\right]$$

This process will be repeated until the error valve becomes acceptable.

### R–S–N relation

In Basquin power law, a log–log straight linear relationship is considered between the applied stress cycles and the number of cycles to failure. R–S–N curves are depicted by determining the fatigue life using the Weibull equation of every data set for each reliability at every specified stress level. Then, regression analysis is done to fit a linear curve to the S–N data.

7 $$\mathrm{log}(N)=A-B\ \mathrm{log}(\Delta \sigma );\ \Delta \sigma \ge \Delta {\sigma }_{\infty }$$

In statistical theory, regression analysis is a process of identifying data trends. Regression analysis commonly uses regression analysis to evaluate relationships between some factors, including a dependent factor and other independent factors (variables). This process shows which variable is essential and which f variable can be ignored. It also shows how these variables affect each other. Linear regression is usually applied to estimate data trends. This method depends on the problem and determines one or more lines to fit the data with minimum error according to a specific mathematical calculation like ordinary least squares. This technique calculates a unique line with a minimum difference between the true data and that line compared to the sum of squared differences. In the statistical analysis of fatigue problem, selecting the best curve fitting method sometimes become very complicated. To avoid uncertainty about a divergent solution, we applied the K–S test to the presented sequences method at the final step.

## Results and discussion

The test data used to evaluate the modified Weibull probability distribution parameters are extracted from the literature^4,36–39^ and presented as six grouped sets of fatigue life data in Table 3. Every data set is a set of fatigue life experimentally measured at same condition and every data group is some data set at different stress levels. C and F data groups have been accomplished only in a single stress level and thus in this study only are applied to modify Weibull parameters. The other data groups in Table 3 are fatigue life scatter at different applied stress levels and are used to modify Weibull parameters and also evaluate stress-life relation for various reliability levels. For numerical solution of the mentioned procedure, a simple M-File code was written in Matlab and Matlab curve fitting tool was applied for regression analysis.

**Table 3:** Test data used to evaluate the modified Weibull parameters.

Data group	Data set	Fatigue life (10^6^ cycles)
A^4^	Data set 1 (S = 4900 MPa)	22.5, 38.8, 64.2, 141, 141, 170, 185, 210, 361, 416, 421, 486, 829, 1390
	Data set 2 (S = 5500 MPa)	8.77, 15, 24.8, 54, 64.9, 65.2, 70.7, 80.2, 137, 158, 160, 184, 312, 520
	Data set 3 (S = 6100 MPa)	5.88, 9.43, 14.6, 29, 34.1, 34.2, 36.7, 41, 65.7, 74.2, 75.1, 85, 135, 211
	Data set 4 (S = 6500 MPa)	1.63, 2.88, 3.72, 4.38, 5.98, 7.99, 13.9, 18.3, 50.7, 57.3, 66.1, 70.7, 103, 179
B^36^	Data set 5 (S = 126 MPa)	0.766126, 0.775688, 1.084235, 1.236622, 1.272766, 1.313096, 1.333748, 1.334498, 1.401002, 1.514865, 1.689579, 1.882968, 1.904087, 1.964597
	Data set 6 (S = 144 MPa)	0.28268, 0.30957, 0.3551, 0.398319, 0.45602, 0.45648, 0.496652, 0.5232, 0.554726, 0.647726, 0.66155, 0.683264, 0.739415, 0.827473, 0.8856, 0.897183, 0.989, 1.20064
	Data set 7 (S = 180 MPa)	0.112302, 0.1278, 0.1335, 0.13371, 0.13384, 0.138322, 0.142491, 0.235782, 0.2898, 0.302732, 0.3303
C^37^	Data set 8 (S = 67.62 MPa)	0.128657, 0.398586, 0.656841, 0.799445
D^38^	Data set 9 (L = 140 mm, S = 46.14 MPa)	8.704, 10.7, 13.17, 13.23, 22.13
	Data set 10 (L = 140 mm, S = 76.96 MPa)	2.33, 2.156, 2.091, 1.25, 1.695
	Data set 11 (L = 140 mm, S = 114.66 MPa)	0.467, 1.16, 0.693, 0.759, 5.67
	Data set 12 (L = 140 mm, S = 160.53 MPa)	0.28, 0.202, 0.214, 0.769
E^39^	Data set 13 (L = 19 mm, S = 53 MPa)	49.2, 49.6, 45, 44.1
	Data set 14 (L = 19 mm, S = 62.5 MPa)	9.3, 13.2, 14, 15.7
	Data set 15 (L = 19 mm, S = 89.5 MPa)	3.87, 3.950, 2.42, 2.82
	Data set 16 (L = 19 mm, S = 132 MPa)	1.22, 1.66, 2.39, 2.66
F^39^	Data set 17 (concrete with steel fiber, V = 1%, S = 0.8 MPa)	0.040135, 0.050139, 0.058141, 0.082345, 0.0984, 0.12862, 0.146544, 0.23916, 0.292488, 0.460482

### Survival probabilities of test data

**Figure 2:**
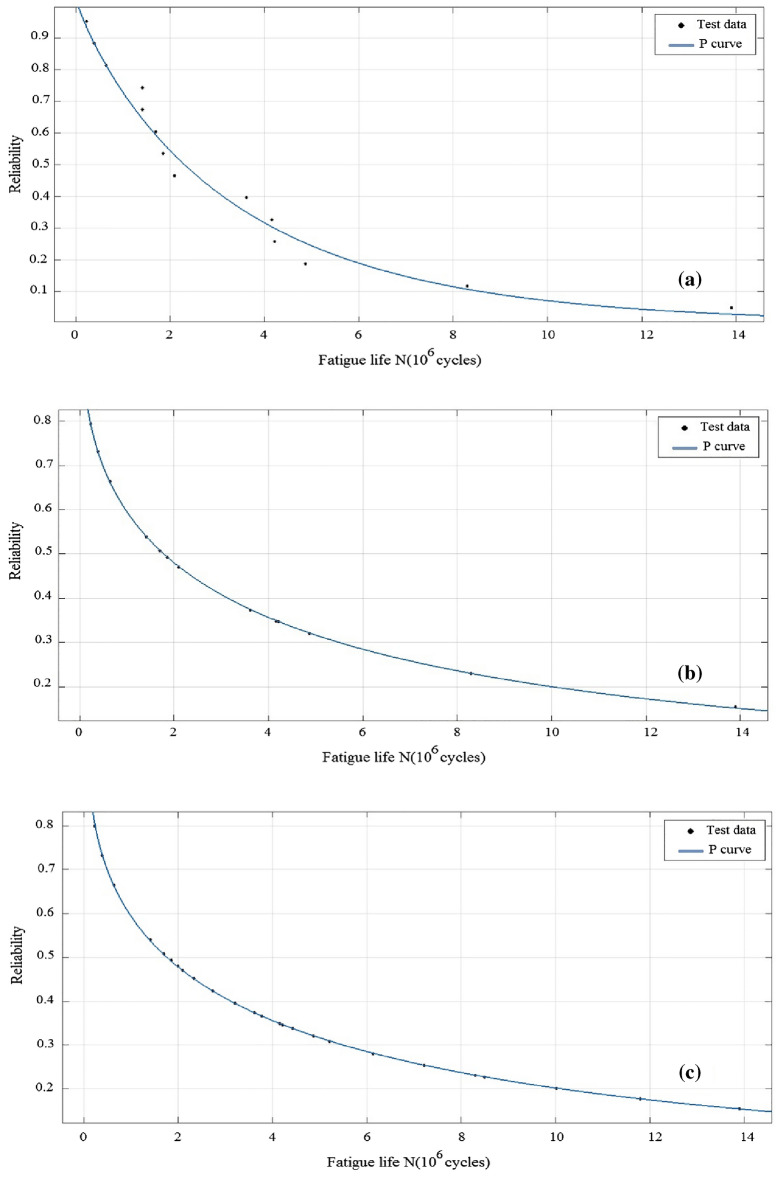
Calculated reliability for (**a**) Common Weibull distribution, (**b**) Modified Weibull distribution and (**c**) Modified Weibull distribution with artificially increase of test data.

The statistical parameters can be determined by repeating the steps of flowchart in Fig. 1. Also, the P-N_f_ (survival probability-fatigue life) relation and Weibull parameters can be evaluated using Eqs. (3), (4c), and (5). Applying regression analysis and curve fitting, Weibull parameters of $b$, $\theta$, and ${N}_{{f}_{0}}$ can be determined in terms of the least square method (Fig. 2). The difference between test results and the distribution model shows good compliance in the modified Weibull model. The effect of sample size and deviation from Weibull distribution was reduced in the test points.

Further, by applying Eqs. (3), (4c), and (5), curve fitting, and determining Weibull parameters, the error between curves and test data sets can be obtained using Eq. (6) for the K–S test and R-square value, respectively. The results of the present modeling are given in Table 4. Here, the value of *θ* in Weibull parameters is almost constant. All the K–S test and R-square values show less error in the modified Weibull model, which complies with curves and test data in Fig. 2.

**Table 4:** Results of modified Weibull parameters.

Test data^4,37–39^	Distribution function	Weibull parameters			Error	
		$b$	$\theta$	${N}_{{f}_{0}}$	K–S test (%)	R-square
Data set 1	Step function	0.89	349,600,000	5,497,000	8	0.986
	Modified Weibull	0.62	344,000,000	9,050,000	6	0.999
Data set 2	Step function	0.90	132,806,849	4,384,680	10.2	0.967
	Modified Weibull	0.62	137,984,686	4,019,219	5.4	0.999
Data set 3	Step function	1	63,647,140	2,938,570	8.7	0.969
	Modified Weibull	0.7	65,883,507	2,160,990	5.2	0.999
Data set 4	Step function	0.58	32,506,615	1,479,600	11.6	0.963
	Modified Weibull	0.4	37,336,316	2,574,857	9.2	0.997
Data set 6	Step function	1.59	602,100	117,100	9.3	0.93
	Modified Weibull	0.86	592,300	79,200	5	1
Data set 8	Step function	0.85	169,100	61,130	16	0.976
	Modified Weibull	0.69	176,000	140,000	2	1
Data set 16	Step function	1.1	179,400	1647	10.2	0.980
	Modified Weibull	0.7	168,000	15,938	5.8	0.993

As shown in Table 4, using the modified method, the error values of the K–S test declined, and the R-square value approached 1. In all cases, the Weibull slope or shape parameter (*b*) decreased. The characteristic life *θ* has changed from 1.5 to 14%, but the minimum time or cycles to failure ${N}_{{f}_{0}}$ has changed considerably up to 10 times. The values of minimum expected cycles to failure obtained from Weibull Distribution in this approach should not be used directly for design.

For the set of rolling contact fatigue life (i.e., data set 1), the smaller fatigue life will have a larger survival probability. Survival probability data can be evaluated using Eqs. (4a) and (5). Figure 2 shows the results of regression analysis and curve fitting. For the test data, reliability of fatigue life is calculated based on the modified and common Weibull distributions. As indicated in Fig. 2, modified Weibull parameters have better compliance between the Weibull model and test results, in the both cases of equal and artificially increased sample size. Here, the effect of the step function for percent of failure and deviation from the Weibull probability distribution function has been eliminated.

**Figure 3:**
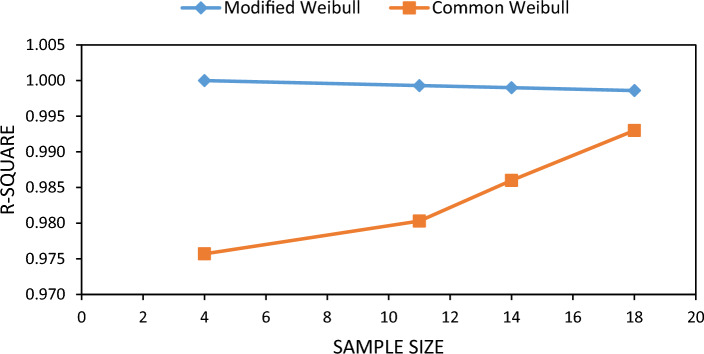
Effect of sample size on the R-Square value for Weibull distribution (data sets 5, 6, 7 and 8).

Figure 3 shows the R-Square values of common Weibull and presented model with respect to sample size. It can be seen that as the sample size increases, the error of the common Weibull model becomes negligible. Small sample size cases had a greater jump in the percent failure function, leading to higher error values.

**Figure 4:**
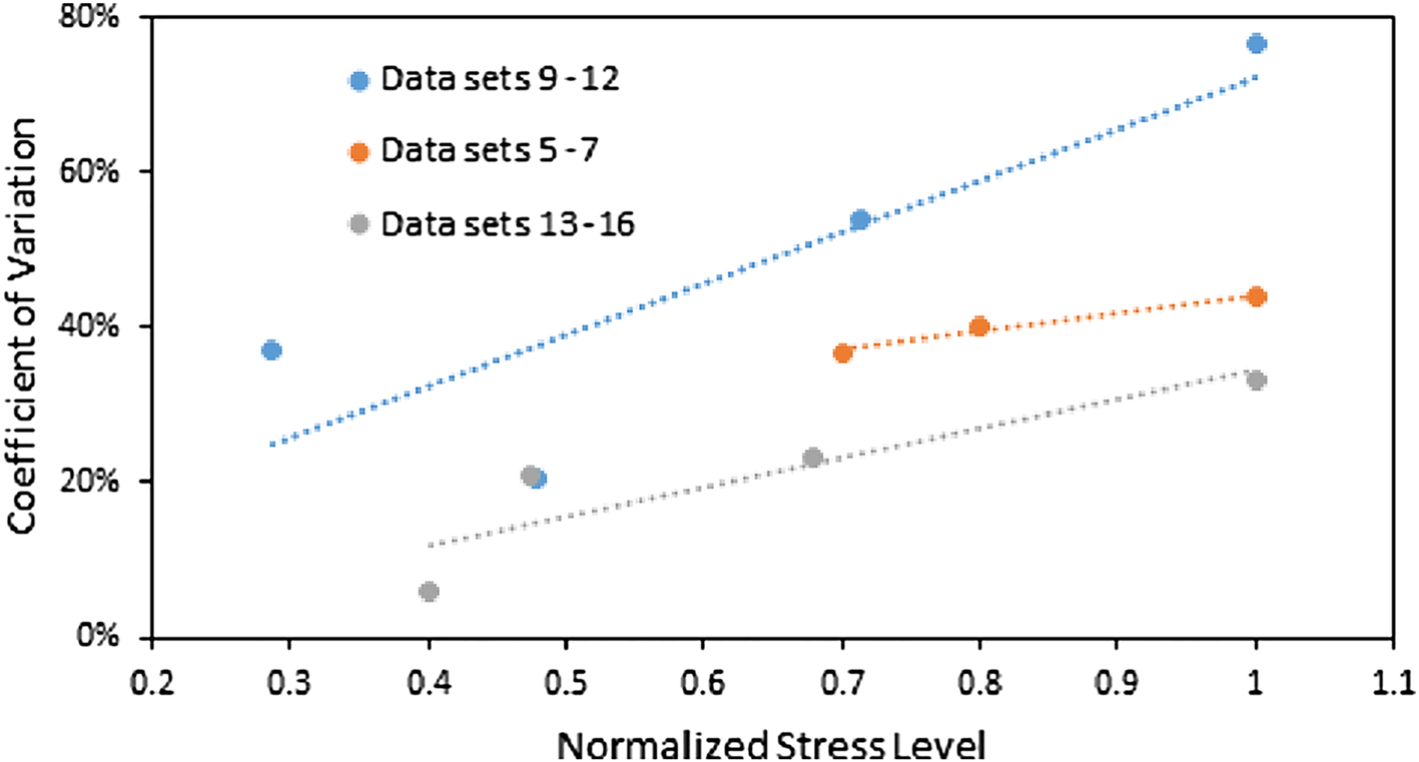
Effect of stress level on the coefficient of variation of fatigue life.

The effect of normalized stress level (with respect to the maximum stress in each data set) on the fatigue life scatter is presented in Fig. 4. As can be seen from Fig. 4, the lower applied stress amplitudes have less data scattering.

### R–S–N relation

**Figure 5:**
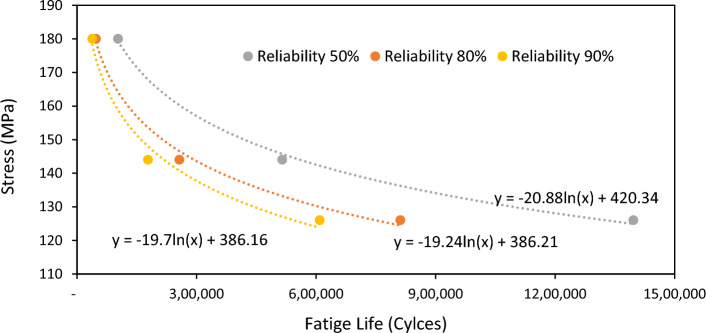
R–S–N curves for 50%, 80%, and 90% reliability.

By applying Eq. (7), Weibull parameters of datasets 5–7 from Table 3, and using regression analysis, Basquin parameters A and B can be determined in terms of the least square method. The S–N curves in Fig. 5 show that for the higher survival probability, the expected fatigue life would decrease at every stress level. Engineering designs usually are in the 0.01 percent probability of failure range^6^. Therefore, extrapolation is required. By extrapolating these curves to the helpful percent probability of failure range, the curves would intersect, which is unreasonable. Also, Table 4 shows that the value of least fatigue life has not reasonable behavior with an increase in the stress level.

**Figure 6:**
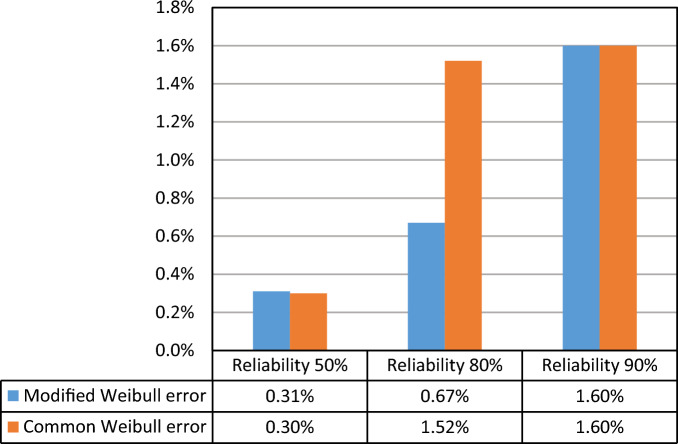
Curve fitting error (1 minus R-Square) in R–S–N curves.

The deviation from the linear relation in the logarithmic scale is calculated considering the Basquin S–N relation and using regression analysis. Figure 6 shows the curve fitting error in the 3 reliability levels. As can be seen, the error increased at a higher reliability level.

## Conclusion

A general method for estimating the parameters of Weibull distribution for modeling general fatigue life data scatter for every applied load and stress region was developed in this study. By considering any result of fatigue test as an equivalent Weibull distribution, artificial data are generated and the accuracy of common Weibull distribution model can be improved. Next, the corresponding fatigue life was evaluated at any reliability using the determined distribution model for any specified stress level. Using Basquin Stress-Life relation and fitting the S–N curves, R–S–N curves were obtained at any reliability level. Overall, the major results of this study can be outlined as follows:

1. The presented method causes the failure percent of test data to increase smoothly and be close to the Weibull distribution curve.
2. Modified coefficients of the distribution function have an acceptable error in the K–S test and R-square value. Also, the difference between test results and probability distribution function was decreased.
3. The effect of discretizing of percent of failure in the sample fatigue life results was decreased, and this approach can be used in all the other small sample size test data. Modifying Weibull parameters has a greater effect on the decreasing error.
4. The fatigue life scatters increase in the higher stress levels.
5. R–S–N curves were obtained using the Basquin Stress-Life relation.
6. The results show the curves cannot be extrapolated to minimum expected life and very high levels of reliability.

Future research(es) may involve using neutrosophic statistics to extend this study. Neutrosophic statistics is the extension of classical statistics and is applied when the data is coming from an uncertain environment like new pandemic or from a complex process like fatigue problems in engineering^40–43^.

## Data Availability

All data generated or analyzed during this study are included in this published article.
